# Artificial molecular motors in biological applications

**DOI:** 10.3389/fmolb.2024.1510619

**Published:** 2025-01-08

**Authors:** Fuli Fan, Songshen Liu, Yuting Yan, Peng Zhang, Kui Che

**Affiliations:** ^1^ Department of Hematology, The Affiliate Hospital of Qingdao University, Qingdao University, Qingdao, China; ^2^ Department of Critical Care Medicine, The Affiliated Hospital of Qingdao University, Qingdao University, Qingdao, China; ^3^ Shandong University of Science and Technology, Qingdao, China; ^4^ Key Laboratory of Thyroid Disease, Medical Research Center, The Affiliated Hospital of Qingdao University, Qingdao University, Qingdao, China

**Keywords:** artificial molecular motor, rotary motion, biological application, cancer, drug delivery

## Abstract

Molecular motors are the cornerstone for the maintenance of living systems and mediate almost all fundamental processes involved in cellular trafficking. The intricate mechanisms underlying natural molecular motors have been elucidated in detail, inspiring researchers in various fields to construct artificial systems with multi-domain applications. This review summarises the characteristics of molecular motors, biomimetic approaches for their design and operation, and recent biological applications.

## 1 Introduction

Natural molecular motors are integral to the proper functioning of living organisms, facilitating fundamental cellular processes through their intricate, evolutionarily honed mechanisms (Tasbas et al., 2021). These motors, such as adenosine triphosphate (ATP) synthase and kinesins, harness chemical energy to drive directional movements, underpinning key biological activities including energy generation and intracellular cargo transport (Schliwa and Woehlke, 2003). By catalyzing the hydrolysis of ATP, these motors efficiently convert chemical energy into mechanical force, enabling muscle contraction, cell division, and gene transcription (Hirokawa et al., 2010; Naydenov et al., 2021).

Molecular motors generally exhibit two types of movements: linear propulsion and rotation. Linear motors, such as myosin, kinesin, DNA helicase, and RNA polymerase, travel directionally along linear tracks to execute their functions (Titu and Gilbert, 1999; Gebhardt et al., 2010; Goulet and Moores, 2013). Rotatory molecular motors, exemplified by ATP synthase, rely on relative motion between a stator and rotor to drive the movement of ions and other substrates (Wilkens, 2005; Kinosita, 1998). Despite their functional diversity, natural molecular motors typically share three fundamental characteristics: (1) pronounced Brownian motion due to their nanometer scale and molecular weight ranges from tens to hundreds of thousands of Daltons (Briane et al., 2020); (2) energy derived from ATP hydrolysis, providing a strong, unbalanced driving force (Kistemaker et al., 2021); and (3) the exhibition of periodic orbital motions during operation (Kolomeisky, 2013).

Inspired by these complex and highly efficient biological systems, researchers have created a variety of artificial molecular motors that can be driven by alternative energy sources. These synthetic motors have been activated using pH, light, and chemical or electrochemical reactions (Bissell et al., 1994; Ashton et al., 1997; Wang et al., 2022), leading to diverse solutions ranging from organic nanoscale motors to larger DNA-based macro-motors (Wickham et al., 2012; Erbas-Cakmak et al., 2015; Kay and Leigh, 2015). In many instances, nanoparticles or nanotubes form part of these systems, creating complex and versatile architectures.

Recent advances include artificial molecular motors constructed from poly (azopeptide) polymers driven by light (Kassem et al., 2017) and DNA-based assemblies that emulate myosin- or kinesin-like stepping motions (Kelly, 2005; Yang et al., 2019). Other strategies have focused on incorporating rotaxanes, catenanes, and fluorenes to achieve rotational motion analogous to natural rotary motors (Harada, 2001; Li et al., 2016; Pan and Liu, 2016; Valero et al., 2018). These artificial motors not only serve as catalysts, transporters, syringes, and pumps but also open new avenues in biotherapeutic applications. As novel agents in cancer treatment, mesenchymal stem cell induction, bactericidal therapy, and drug delivery, artificial molecular motors hold great promise for biomedical interventions. In the following sections, we will discuss the design, mechanisms, and biotherapeutic applications of artificial molecular motors, providing a comprehensive overview of their current status and potential future directions.

## 2 Biological applications of artificial molecular motors based on transmembrane transport

Natural molecular motors, such as ATP-driven pumps and rotary motors, actively convert chemical energy into mechanical work to transport ions and molecules across lipid membranes. Inspired by these complex biological systems, researchers have developed artificial molecular motors that also harness and convert external energy sources—such as light, chemical fuels, or electrochemical gradients—into directed motion, enabling controlled transmembrane transport.

Unlike passive channels or pores that rely solely on concentration gradients or membrane potentials to move cargoes, these active synthetic motors undergo conformational changes upon energy input, thereby opening or closing transmembrane pathways with high specificity and temporal precision. One representative example involves the integration of light-responsive groups into channel proteins. By conjugating photo-sensitive cyanine moieties to membrane proteins, researchers have devised artificial channels whose conformations—and thus permeability—can be reversibly modulated by UV irradiation (Koçer et al., 2005; Ribovski et al., 2020). Under illumination, large-scale protein rearrangements occur, allowing target molecules or ions to move across the membrane. When the light source is removed, the channels return to their initial closed state, restoring membrane integrity. Such reversible, light-gated systems illustrate the principle of using external energy to directly govern transmembrane transport in a controlled manner.

Recent advancements have further refined these constructs, achieving enhanced selectivity, improved switching frequencies, and compatibility with near-infrared (NIR) or visible light sources. These improvements facilitate deeper tissue penetration and reduced phototoxicity, broadening the biomedical utility of active molecular motors. For instance, some designs integrate chemical fuel inputs to drive repetitive conformational cycles, simulating the directional and autonomous pumping found in natural pumps, yet with customizable responses and operational conditions. Such systems can potentially serve as precision drug delivery platforms, allowing therapeutic agents to be released only when and where needed, thereby improving treatment efficacy and reducing systemic side effects.

By emphasizing synthetic molecular motors that actively convert energy into mechanical work, the focus remains on truly dynamic and controllable transmembrane transport. The resulting systems advance beyond passive diffusion mechanisms, paving the way for smart, adaptable bio-inspired technologies that hold significant promise for next-generation therapeutics, nanoscale devices, and engineered biomimetic interfaces.

## 3 Anti-cancer treatment of artificial molecular motors based on drug-delivery systems

**FIGURE 1:**
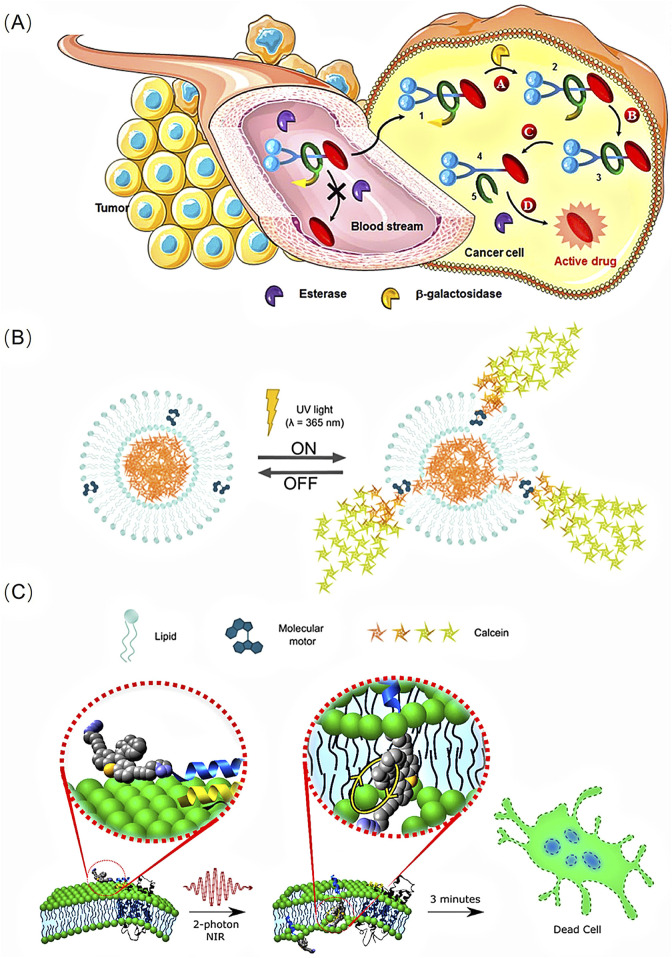
**(A)**The principle of the intracellular drug delivery with functional interlocked enzyme-sensitive [2]-rotaxane 1. Reproduced from (Barat et al., 2015) with permission from The Royal Society of Chemistry. **(B)** Schematic representation of the mode of operation of on demand release from liposomes with molecular motors incorporated in the lipid bilayer. Reproduced from (Ribovski et al., 2020) with permission from The Royal Society of Chemistry. **(C)** Schematic of a molecular machine selectively binding to the membrane surface through a peptide recognition and then drilling through a cell membrane by NIR 2 PE-activated molecular mechanical action to kill the cell. Reproduced from (Liu et al., 2019) with permission from American Chemical Society.

Recently, rotaxane- and pseudorotaxane-based molecular motors have emerged as efficient delivery systems for anticancer drugs. Notably, enzyme-sensitive [2]-rotaxane 1 has been reported to release a potent anticancer drug within tumour cells (Barat et al., 2015). To obtain a biocompatible autonomous system, researchers designed a rotaxane 1, incorporating an enzymatic trigger (β-galactosidase), a self-opening macrocycle, and various linkers or stoppers. The presence of a macrocycle stabilises the drug in the bloodstream. Once inside KB cells (human mouth epidermal carcinoma), the activation of the enzymatic trigger initiates a series of chemical reactions, ultimately leading to the decomposition of the interlocked architecture and the release of the anticancer drug paclitaxel within the cells (Figure 1A).

A key aspect of drug delivery systems is not only to accommodate the agents but also to release them on demand, thereby increasing local concentrations to enhance treatment efficiency and reduce side effects. Researchers have proposed a molecular motor-liposome complex that enables light-induced molecular rotation and triggers on-demand release from liposomes (Ribovski et al., 2020). The artificial molecular motor used in this system is a hydrophobic unidirectional rotary motor which remains embedded within the lipid bilayer and opens the liposome membrane to release small drug molecules (such as calcein) when irradiated with 365 nm light. This release occurs only in the presence of the molecular motor combined with UV irradiation (Figure 1B).

Recent advances in this field have expanded the capabilities of molecular motor-based systems. For instance, “motile-targeting” micro/nanorobots have been developed, enabling precise delivery of anti-cancer drugs like doxorubicin directly into tumor tissues. These systems utilize their motility to cross biological barriers and actively accumulate at tumor sites, significantly enhancing therapeutic outcomes (Zhang et al., 2022). Moreover, catalytic nanorobots functionalized with paclitaxel and phenylboronic acid have demonstrated efficient drug delivery and controlled release mechanisms, further optimizing the pharmacokinetics of anticancer agents (Demirbuken et al., 2022). Another innovative approach involves dual-functional nanomotors capable of photothermal therapy combined with drug delivery. These systems incorporate biocompatible scaffolds for simultaneous transportation of chemotherapeutic and photothermal agents, providing synergistic effects for tumor ablation (Li et al., 2021). Collectively, these advancements highlight the transformative potential of molecular motor-based drug delivery systems in overcoming challenges associated with conventional therapies, offering precise, efficient, and minimally invasive solutions.

## 4 Anti-cancer treatment of artificial molecular motors based on cell membrane permeabilization

An elaborately designed light-activated molecular motor has been demonstrated to open cellular membranes, thereby expediting cell death (García-López et al., 2017). To mitigate the UV-induced damage, researchers have developed a less destructive two-photon excitation (2 PE) method using NIR light activation. The designed rotor molecular nanomachines can be targeted to specific cell surfaces through peptide recognition, enabling single-cell death with optical precision. Once triggered by NIR light through 2 PE at wavelengths of 710–720 nm in a three-dimensional (3D) raster pattern, the molecular machines can specifically and selectively penetrate through the cell membrane and kill the cell (Figure 1C). Additionally, *in vitro* experiments have verified the killing effect on various cancer cell lines, including PC3 (human prostate cancer), HeLa (cervical cancer), and MCF7 (human breast cancer) cells (Liu et al., 2019).

Recent studies have further demonstrated the versatility of light-activated molecular motors in cancer treatment. For instance, molecular motors functionalized with photosensitizers have been shown to enhance photodynamic therapy (PDT) by generating reactive oxygen species (ROS) upon light activation, resulting in augmented cancer cell death (Wang et al., 2022). Furthermore, visible light-absorbing synthetic molecular motors have been developed, which can kill pancreatic cancer cells in response to light (Ayala Orozco et al., 2020). The evidence presented supports the conclusion that this type of rotating unidirectional molecular nanomachine can suppress the formation of reactive oxygen species and generate nanomechanical actions to kill cancer cells by drilling holes in cell membranes. This advancement broadens the scope of molecular motors in anti-cancer strategies by introducing highly efficient and less invasive approaches.

In addition, living cell-derived intelligent nanobots have been shown to integrate with cell membranes, achieving enhanced permeability and retention (EPR) effects, which improve targeted drug delivery to tumor cells (Zhou et al., 2024). Micro/nanobots with motile-targeting capabilities have been engineered to carry anti-cancer drugs, effectively fusing with cancer cell membranes to deliver therapeutic agents (Zhang et al., 2022). Nano-and micromotors have also been highlighted for their ability to disrupt cancer cell membranes, offering a non-invasive yet precise approach to cancer treatment (Sonntag et al., 2019). Furthermore, molecular motors equipped with light-controlled logic-gated K+ channels have demonstrated their capability to selectively induce cancer cell apoptosis through mechanisms involving cell membrane permeabilization and ion transport (Li et al., 2024).

These advancements collectively underscore the transformative potential of molecular motors in oncology, paving the way for novel therapeutic strategies that combine high precision, minimal invasiveness, and enhanced efficacy in cancer treatment.

## 5 Artificial molecular motors for antimicrobial treatment

Multidrug-resistant pathogenic bacteria pose an increasingly serious challenge to anti-infective therapy and preventative practices. With approximately 700,000 deaths attributed to multidrug-resistant pathogenic bacteria or strains annually (O’Neill, 2016), researchers have proposed that synthetic molecular motors could serve as an effective treatment for killing drug-resistant pathogens. This strategy is based on the hypothesis that molecular nanomachines can penetrate bacterial cell walls and increase their susceptibility to antibiotics.

*Klebsiella pneumoniae* can cause various healthcare-associated infections, including pneumonia, bloodstream infections, urinary tract infections, wound or surgical-site infections, and meningitis. Recently, a type of artificial molecular motor with a rotor component has been developed that is actuated with 365 nm light, enabling unidirectional rotation relative to a stator. By rapidly rotating, these molecular nanomachines can disrupt the inner and outer cell walls of *K. pneumoniae*. When combined with antibiotic pharmacology studies, carbapenem antibiotics like meropenem demonstrate bactericidal effects in penicillin-binding proteins that inhibit cell wall synthesis. Therefore, the disruption of the outer cell wall membrane by the above-mentioned molecular motors could synergize with the currently ineffective carbapenem antibiotics and make them more effective for anti-infective therapy (Galbadage et al., 2019).

In addition to killing multidrug-resistant bacteria, studies have shown that artificial molecular motor can effectively eliminate parasites. These studies have also shown that artificial molecular motors can disrupt cell membranes *in vivo*. These findings suggest that molecular motors can rotate rapidly upon activation with 365 nm light. This rotation causes pathological changes and mortality in *Caenorhabditis elegans*, which is a multicellular organism (Gunasekera et al., 2020).

## 6 Artificial molecular motors for mesenchymal stem cells differentiation

Dynamic physicochemical and mechanostructural changes can regulate and control the behaviour of cells in space and time. In other words, molecular motion is fatal to the development and cellular processes (Li et al., 2017). Such pivotal characteristics also crucial for tissue repair and regeneration.

**FIGURE 2:**
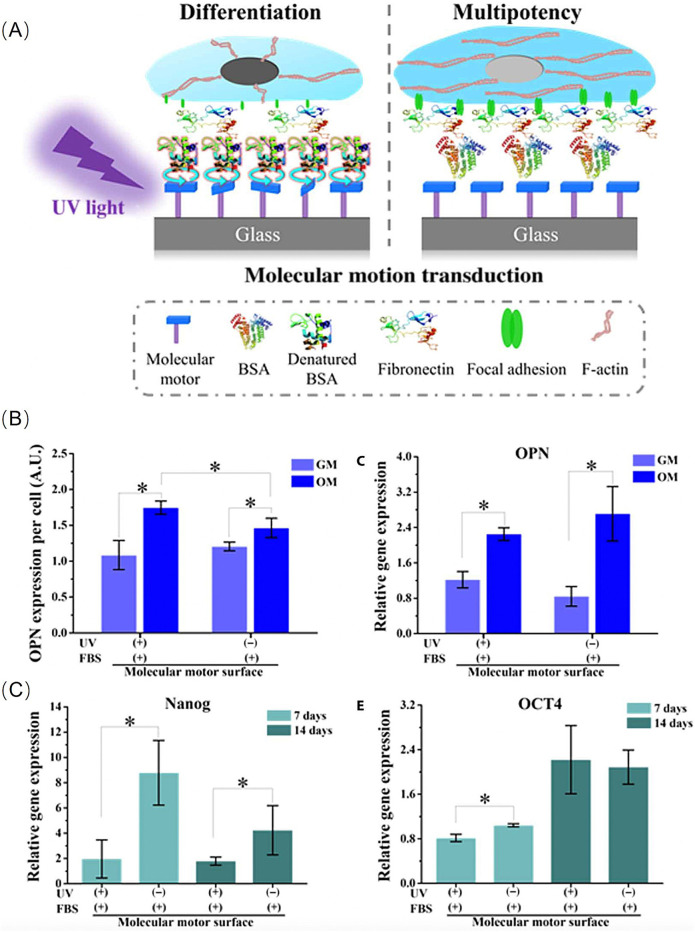
**(A)**Schematic illustration of the molecular motion directing the differentiation of hBM-MSCs. **(B)** Protein level and Gene level: Quantification of the expression of OPN. **(C)** RT-PCR analysis of relative mRNA expression level of Nanog and OCT4 genes at different time points. Reproduced from (Zhou et al., 2020) with permission from Science Advance.

Inspired by the properties of biomolecular-motors, researchers have developed ideas for designing surface-bound molecular-motors that mediate adsorbed proteins, and subsequently direct the fate of stem cells (Zhou et al., 2020). In this construction, light-driven unidirectional rotary motion of the motor is confined to the amine-modified glass surfaces through electrostatic interactions. Next, under UV irradiation (λmax = 365 nm), bovine serum albumin (BSA) from foetal bovine serum was adsorbed onto the motor’s surface and continuously cultured for 1 h. Human bone marrow-derived mesenchymal stem cells (hBM-MSCs) were seeded on the treated motor surfaces to study cell adhesion, proliferation, differentiation, and maintenance of function (Figure 2A).

Unidirectional rotating molecular-motors can increase BSA adsorption and influence the FA cytoskeleton actin transduction pathway, as well as macroscopic cell adhesion and morphology. Thus, this synthetic artificial motor could mediate the fate of hBM-MSCs. The experimental results indicated that stem cells on this dynamically altered surface were more likely to differentiate into osteoblasts, whereas without UV irradiation on the static surface, they tended to maintain their function as stem cells (Figures 2B, C).

Another elaborate photo-actuating motor design that mimics native extracellular matrix (ECM) as an hBM-MSC culture scaffold has been developed (Chen et al., 2022). This article presents supramolecular assemblies formed by motor amphiphiles (MAs), which are based on a second-generation molecular-motor core, combined with a hydrophobic alkyl chain and hydrophilic chains with various end-groups, including carboxylate, phosphite, and sulfate groups. Under UV irradiation (λmax = 365 nm), this study also demonstrated that MAs do not merely indicate no toxicity and high biocompatibility of hBM-MSCs, but also the possibility of good cell proliferation. In particular, the MAs demonstrated photoenergy conversion into mechanical actuation in the presence of hBM-MSCs.

## 7 Artificial molecular motors for tumor nanotheranostics

To locate tumours, ascertain their progression, or gauge the response to therapy, various imaging methods, including X-ray computed tomography (CT), magnetic resonance imaging (MRI), and positron emission tomography (PET), are routinely applied. In the past few years, artificial molecular motors with diagnostic or imaging capabilities for tumour diagnosis have attracted significant interest from researchers. Derived from the different microenvironments surrounding the tumour in terms of pH and enzymatic expression, distinct environmental conditions can trigger tumour-specific molecular motion of artificial molecular motors. The distance between the wheel and stopper in rotaxanes, as well as the relative positions of the locked rings in catenanes, can be adjusted in response to these external microenvironments (Yu et al., 2018). For example, the rotaxanes and catenanes developed by Sauvage et al., which contain bidentate and terdentate ligands, serve as potential chelators of transition metals for MRI and PET imaging (Collin et al., 2001).

Recent advancements have expanded the scope of molecular motors in nanotheranostics. For instance, nanomotors functionalized with upconversion nanoparticles (UCNPs) have been developed for tumor imaging and multimodal synergistic therapy, integrating photothermal and photodynamic effects for enhanced therapeutic efficacy (Liu et al., 2022). Additionally, artificial molecular machines capable of light-stimulated motion have been utilized to improve tumor targeting, enabling precise delivery and activation of therapeutic agents within tumor microenvironments (Thorat et al., 2019). Furthermore, dual drug-loaded calabash-shaped nanomotors have demonstrated exceptional performance in chemo-photothermal therapy, particularly in the treatment of orthotopic glioblastomas, due to their active targeting and dual-mode therapeutic action (Li et al., 2023). Advances in fluorescence imaging have also enabled the application of artificial molecular motors as light-responsive probes, facilitating real-time visualization of tumor progression and therapeutic response (Yang et al., 2023).

These studies highlight the transformative potential of artificial molecular motors in tumor nanotheranostics, paving the way for precise, minimally invasive, and multimodal cancer diagnostics and therapies.

## 8 Advances in light-driven synthetic molecular motors

In recent years, the development of light-driven molecular motors has significantly advanced the fields of nanotechnology and smart materials. These molecular motors harness light energy to induce mechanical motion, enabling precise control over molecular-level dynamics. A notable innovation involves the design of all-visible-light-driven molecular motors, which enhance biocompatibility while mitigating UV-related damage. For example, Schiff-base-functionalized motors have demonstrated optimized thermodynamic properties and rotational kinetics, allowing efficient operation in complex chemical environments (van Vliet et al., 2024).

Furthermore, light-driven molecular motors have been integrated into three-dimensional liquid crystal elastomers to create biomimetic functions. Using advanced 3D printing technologies, researchers have embedded molecular motors within liquid crystal networks, enabling the controlled actuation of materials that mimic natural biological systems. This approach not only expands the functional applications of molecular motors but also paves the way for innovations in responsive materials and soft robotics (Long et al., 2024).

Additionally, supramolecular systems utilizing light-driven molecular motors have been explored for their ability to induce polymerization and form complex assemblies. Amphiphilic designs have been particularly effective in leveraging the rotational motion of molecular motors to drive the formation of hierarchical structures, showcasing potential in self-assembly and material synthesis (Schiel et al., 2024). These advancements illustrate the diversity and versatility of light-driven molecular motors, underscoring their transformative potential in the development of next-generation dynamic materials.

## 9 Discussion

The 2016 Nobel Prize in Chemistry was awarded to Professors Bernard Feringa, Jean-Pierre Sauvage, and Sir James Fraser Stoddart for their groundbreaking contributions to the design and development of molecular machines. While these advancements marked a pivotal moment for the field, artificial molecular motors remain in their nascent stages, requiring further exploration and innovation. This review summarized the principles of operation and biological applications of artificial molecular motors, particularly in the contexts of cancer treatment, theranostics, antimicrobial therapy, and stem cell differentiation.

Compared to traditional biological materials, molecular motors offer unique advantages. Their non-toxic nature, coupled with their ability to perform targeted motion, makes them a promising tool for precision medicine. These motors can be selectively activated within cancer cells, sparing healthy tissues from collateral damage. For instance, drug delivery systems based on molecular motors enable the efficient loading of therapeutic agents, diagnostic molecules, and targeting ligands, minimizing the reliance on time-consuming and expensive covalent synthesis methods.

Despite these advantages, significant challenges remain. The optimization of molecular motor conformations and functions to enhance their biological applications is still an area of active research. Current molecular motors are predominantly driven by stimuli such as pH changes or specific wavelengths of light, which may limit their versatility in complex biological environments. Recent innovations, such as all-visible-light-driven molecular motors and systems incorporating biomimetic functions, have expanded their operational scope, but the exploration of novel stimuli, such as magnetic fields, acoustic waves, or enzymatic triggers, could further enhance their applicability and performance.

The integration of artificial molecular motors into advanced therapeutic strategies has demonstrated their potential to revolutionize disease treatment. For example, in cancer therapy, dual-functional nanomotors combining photothermal and chemotherapeutic effects have shown remarkable efficacy, particularly for aggressive tumors such as glioblastomas. Similarly, the application of motile-targeting micro/nanorobots has enhanced drug delivery precision, overcoming barriers posed by tumor microenvironments. These advancements underscore the transformative role of molecular motors in the development of supramolecular nanomedicines.

Artificial molecular motors also exhibit promise beyond oncology. For instance, their utility in antimicrobial treatments demonstrates their versatility, with light-activated molecular motors effectively disrupting bacterial membranes and enhancing antibiotic efficacy. Moreover, their application in supramolecular polymerization and material assembly highlights their potential in fields ranging from tissue engineering to responsive materials.

Looking forward, artificial molecular motors are poised to play a critical role in the treatment of numerous diseases. Continued research into their design and biological properties will enable the creation of versatile, stimuli-responsive systems with unparalleled specificity and efficacy. With significant interdisciplinary efforts and advances in supramolecular chemistry, we are optimistic that nanomedicines based on artificial molecular motors will provide innovative solutions and renewed hope for patients battling complex and challenging diseases.
